# Anterior and Posterior Occlusal Plane Inclinations Differ Between Class II and Class III Mixed Dentitions—A Retrospective Cross-Sectional Study of a Morphological Characteristic

**DOI:** 10.3390/jcm14186553

**Published:** 2025-09-17

**Authors:** Aleš Čelar, Stefan Lettner, Erwin Jonke

**Affiliations:** 1 Clinical Division of Orthodontics, University Clinic of Dentistry, Medical University of Vienna, Sensengasse 2a, 1090 Wien, Austria; erwin.jonke@meduniwien.ac.at; 2 Karl Donath Laboratory, University Clinic of Dentistry, Medical University of Vienna, Sensengasse 2a, 1090 Wien, Austria; stefan.lettner@meduniwien.ac.at

**Keywords:** anterior occlusal plane, posterior occlusal plane, cephalometry, mixed dentition, class II, class III malocclusion

## Abstract

**Background:** To investigate if the sagittal inclinations of anterior occlusal plane (AOP) and posterior occlusal plane (POP) differ between skeletal classes II and III in the mixed dentition period. **Methods:** Retrospective analysis of cross-sectional lateral cephalometric radiograph data of 61 children with skeletal class II and 60 children with skeletal class III (average age 8.4 ± 1.5 years). We measured the inclinations of AOP and POP to the Frankfort horizontal (FH) and the sella-nasion line (SN). Angles FH–AOP, FH–POP, SN–AOP, and SN–POP were compared between both groups using model-based ANOVA F tests and quantile regressions. **Results:** The differences in means between the groups came to 1.3° ± 5.1 (FH–AOP), 1.8° ± 4.6 (FH–POP), 2.6° ± 5.3 (SN–AOP), and 5.5° ± 4.4 (SN–POP). In the ANOVA, angles SN–AOP, FH–POP, and SN–POP differed significantly between the groups (*p* = 0.041, *p* = 0.006, *p* < 0.001, respectively). Quantile regressions showed significant between-group differences for FH–AOP (lower quartile, *p* = 0.012), FH–POP (upper quartile, *p* = 0.006), SN–AOP (median, *p* = 0.004; upper quartile, *p* = 0.011), and SN–POP (all 3 quartiles, *p* < 0.001). **Conclusions:** Distinct occlusal plane inclinations of mixed dentitions represent diagnostic traits. Longitudinal and interventional data are needed if therapeutic flattening of mixed dentition AOP or POP is beneficial in treating skeletal class II, same as their steepening in skeletal class III. Our clinical hypothesis suggests alterations by approximately 3° (AOP) and 6° (POP) but requires further study and confirmation.

## 1. Background

The sagittal orientation of the occlusal plane (OP) connotes diagnostic implications beyond dental arch aesthetics. Björk attributed diminished OP inclinations to maxillary or mandibular prognathism [1]. The angle between OP and mandibular plane showed a significant relation to overbite and facial type [2]. Later research identified almost horizontal sagittal inclinations of the OP in class III facial patterns [3] while class II dentitions showed a pronounced posteriorly upward orientation of OP [4]. Moreover, deep overbite, enlarged overjet, and class II malocclusion correlated with an increased curve of Spee [5].

Division of the sagittal maxillary OP into anterior occlusal plane (AOP) and posterior occlusal plane (POP) corroborated steeper AOPs and/or POPs in class II than in class III permanent dentitions [4,6,7,8]. Except one study, [8], these investigations reported the differences in means only and disregarded potential statistical significance of data across the distribution. Hence, quantile regressions become essential to thoroughly comprehend how AOP or POP inclinations affect malocclusion samples beyond the average.

The association between AOP or POP inclinations and class II or class III malocclusion has not been studied widely in mixed dentitions. A single investigation evaluated lateral cephalometric data of the maxillary AOP and POP inclinations in children and adolescents by means of ANOVA [6]. The statistical means of the angles between Frankfort horizontal (FH) and AOP or POP did not differ significantly between the class II and class III groups in children aged 3–10 years.

As significant difference in data is not only related to means, the present study aimed to evaluate AOP and POP inclinations in children with mixed dentitions and class II or class III malocclusions under consideration of the 0.1–0.9 quantiles, including mean, median (Q_2_), and the 0.25 and 0.75 quartiles (Q_1_, Q_3_). The objective was to determine whether or not the sagittal inclinations of AOP or POP differ between skeletal class II and skeletal class III configurations in the mixed dentition period. Our *null hypothesis* stated no significant differences in AOP and POP inclinations between skeletal classes II and III unless proven different.

As the cephalometric delineation of FH can be demanding when the identification of porion or orbitale is difficult [9,10,11], we considered the more reliable sella-nasion line (SN) in addition to FH for measuring the AOP and POP inclinations [12].

## 2. Methods

We followed the STROBE guideline for reporting this retrospective study, which was approved by the institutional review board in accordance with the principles for medical research in humans (Ethics Commission of the Medical University of Vienna, Austria, ID: EK Nr.1187/2012). We investigated anonymized pre-treatment orthodontic records of Caucasian children, whose parents had given informed consent for diagnostic records taken from 2008 to 2022. Eligible participants were recruited from a single university orthodontic department (Medical University of Vienna, University Clinic of Dentistry, Clinical Division of Orthodontics). Patients and their parents or legal guardians came on own their initiative or after referral by their dentist. The period of recruitment and the collection of data took 18 months.

We tried to reduce potential confounders and effect modifiers by the following criteria of eligibility. Inclusion criteria encompassed girls or boys aged <12 years, fully dentate mixed dentitions, and complete eruption of the permanent central incisors and permanent first molars. Exclusion criteria covered congenital anomalies such as cleft lip and palate, craniofacial syndromes (e.g., Apert, Crouzon, Goldenhar, Down, Turner, Gorlin–Goltz, cleidocranial dysostosis, cherubism), extractions of primary and/or permanent teeth, extensive caries with pulp involvement, hypodontia, oligodontia, juvenile idiopathic arthritis, odontoma, history of facial trauma, previous orthodontic treatment, open-bite malocclusion, long-term medication, and missing data.

**Figure 1:**
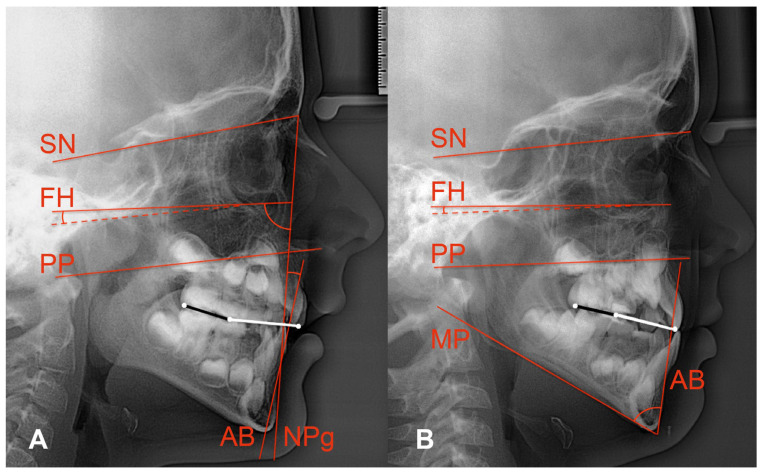
Anterior occlusal plane (white line) and posterior occlusal plane (black line) depicted for mixed dentitions of 2 non-participants showing skeletal class II (**A**) and skeletal class III (**B**) relationships. White occlusal dots show AOP and POP landmarks (maxillary incisor’s edge, occlusal midpoint of maxillary second deciduous molar, distal cusp of averaged permanent first molar). Red lines show SN and FH. (**A**) also illustrates the APDI: sum of the angles between Frankfort horizontal (FH) and nasion-pogonion line (NPg) plus/minus angle between FH and palatal plane (PP; negative signs in both examples) plus/minus angle between NPg and A–B line (AB; negative sign in (**A**)). (**B**) shows the ODI: sum of the angle between AB and the mandibular plane (MP) plus/minus angle between FH and PP.

Diagnostic criteria comprised skeletal class II or skeletal class III as obtained from cephalometric radiographs. Same as Tanaka and Sato [6], we sampled our study groups according to the anteroposterior dysplasia indicator (APDI) [13]: class II when APDI < 77.5, class III when APDI > 85 (Figure 1A).

### 2.1. Sample

Of approximately 1100 potentially eligible children with complete orthodontic records taken from 2008 to 2022, 793 were confirmed eligible (APDI < 77.5 or >85, Europeans, met inclusion and exclusion criteria). We included and analysed 121 individuals after random sampling by systematic selection of anonymized participants from the 15 annual patient lists. We enrolled 4 individuals with class II and another 4 with class III consecutively from each year (and by chance a 5th class II subject once). This approach yielded 55 boys and 66 girls aged 5.6–11.7 years (mean 8.4 ± 1.5). Sixty-one children showed a skeletal class II (36♀︎, 25♂︎) and 60 a skeletal class III (30♀, 30♂). All pre-treatment lateral cephalometric radiographs had been taken in full dental intercuspation with a digital x-ray machine (Philips Bucky Diagnost VS, Philips Healthcare, Eindhoven, The Netherlands; tube-sensor distance 4 m, dose area products 3–5 cGycm^2^, 66–70 kV, 25–40 mAs). Fujifilm FCR XG-1 visualised the digital images (Fuji Photo Film Co., Ltd., Tokyo, Japan).

For determination of the sample size, we used AOP and POP inclinations referenced to FH only because SN-related inclinations were not available in earlier scientific literature. We pooled the standard deviations (SDs) of Tanaka and Sato’s FH–AOP for the ages 6–7 years and 8–10 years, same as the SDs of FH–POP of the same age groups [6]. Multiple comparisons and R^2^ were not considered for sample size calculations. The result showed that 32 subjects per group will distinguish AOP group differences of 3° with a power of 80% when ⍺ = 0.05. For POP, this computation yielded 43 individuals per group.

Efforts to address potential sources of bias were training of the investigators, blinding, use of a standardised protocol, standardised instruments, control of entered data regarding typing errors, and calibration. Two operators performed calibration training for cephalometric landmark identification and measurements on 20 radiographs of non-participants. Both investigators traced the radiographs manually using pencils with 0.5 mm lead width in a silent room with dimmed light. One researcher had 25 years of experience in cephalometric analysis and another researcher had 1 year of practice. Thus, all measurements recorded by the latter researcher were controlled by the former, more experienced investigator.

### 2.2. Cephalometric Landmarks, AOP, POP

We located SN from the centre of the pituitary fossa to nasion, FH from averaged orbitale to averaged porion, palatal plane (PP) from anterior nasal spine to posterior nasal spine, mandibular plane (MP) from menton to averaged tangential gonion, the nasion-pogonion line (NPg), the line from point A to point B (AB), the maxillary incisor, and the averaged molar outlines (Figure 1).

As the original definitions of AOP and POP referred to permanent maxillary incisors, second premolars, and permanent second molars [4], we had to modify the delineations of AOP and POP for mixed dentitions: AOP connected the incisal edge of the maxillary central incisor and the midpoint between the cusp tips of the averaged maxillary deciduous second molars. The latter point and the distal cusp tip of the averaged permanent maxillary first molar defined the mixed dentition POP (Figure 1).

The cephalometric measurements included APDI, Wits appraisal, angles FH–AOP, FH–POP, SN–AOP, SN–POP, and the overbite depth indicator (ODI), a descriptor of vertical skeletal relationships [14]. The ODI adds the angle between FH and PP plus the angle between AB and MP. Angle FH–PP is read positive if PP slopes more forward–downward than FH. Figure 1B shows backward–downward inclined PPs, i.e., negative FH–PP angles. The ODI’s reference range is 74.5 ± 6.1 [14]. Six- to ten-year-old children from the Burlington growth study showed ODI means from 69.3 to 74.3. The greater the ODI, the more skeletal hypodivergence exists, and vice versa.

The operators performed the measurements blinded to the skeletal classification.

### 2.3. Statistics

The reliability of cephalometric data was assessed with intraclass correlation coefficients (ICCs) [15] and 95% limits of agreement [16]. Each operator retraced 25 radiographs selected at random. Manual retracing and second set of measurements were carried out two weeks after the initial tracing.

The normality of data was tested with Q–Q plots. Inferential statistics comprised Pearson correlations, ANOVA F-tests, and quantile regression models for assessment of significant effects at Q_1_, Q_2_, Q_3_ using FH–AOP and FH–POP as dependent variables [17,18]. Age, gender, skeletal class, and ODI were included in the statistical models as covariates and considered as potential confounders. The resulting model formula was Q*_(τ__)_*(*Angle*) = *β_(τ__)0_* + *β_(τ__)1_*(*Skeletal Class*) + *β_(τ__)2_*(*Age*) + *β_(τ__)3_*(*Gender*) + *β_(τ__)4_*(*ODI*). As we aimed to evaluate the association between sagittal malocclusion and AOP and POP inclinations, a powerful vertical parameter as the ODI appeared necessary. Age and gender are common potential confounders. Covariates were integrated in ordinary least squares ANOVA tests. Potential interactions between coefficients were close to zero and not further considered in this paper. Inversion of rank tests yielded the confidence intervals. *P*-values of the statistical model refer to Wald tests (⍺ = 0.05) using Huber sandwich estimates for the standard error [17,19]. Quantile regressions were plotted for the 0.1–0.9 quantiles and re-calculated after replacing FH–AOP and FH–POP by SN–AOP and SN–POP. We computed the quantile regressions in an ordinary least squares-based ANOVA to assess sensitivity to assumptions. Estimates not adjusted by covariates (age, gender, ODI) are available in the Supplementary Materials. Missing data were a criterion of exclusion and did not have to be addressed statistically. We used R software version 4.4.1 for statistical computing and graphics (R Core Team 2024, R: A Language and Environment for Statistical Computing, R Foundation, Vienna, Austria).

## 3. Results

Calculation of the intra-operator ICCs yielded 0.91 for APDI, 0.91 for FH–AOP, 0.92 for FH–POP, 0.95 for ODI, 0.96 for SN–AOP, and 0.96 for SN–POP. The ICCs for the inter-rater reliability of these measurements ranged from 0.85 to 0.92. The Bland–Altman 95% limits of agreement were −2.2 to 1.5 for APDI, −3.2 to 2.1 for FH–AOP, −3.7 to 4 for FH–POP, −3.4 to 3 for ODI, −1.7 to 1.4 for SN–AOP, and −1.6 to 2.4 for SN–POP.

**Figure 2:**
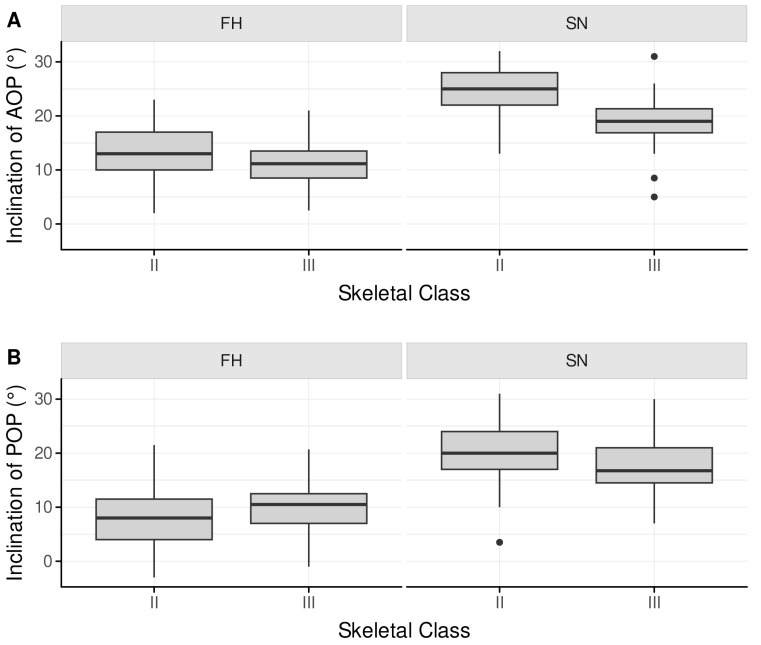
Box plots showing minima, quartiles, and maxima of the measurements FH–AOP, SN–AOP, FH–POP, and SN–POP for the class II and the class III groups. (**A**) FH-AOP and SN-AOP for skeletal classes II and III; (**B**) FH-POP and SN-POP for skeletal classes II and III. Whiskers, box, and median indicate symmetry and skewness; outliers are plotted as dots.

The Q–Q plots showed normality of age, gender, APDI, ODI, Wits appraisal, AOP and POP data. Table 1 presents descriptive statistics of 121 individuals without missing data. Figure 2 shows the box plots for both groups.

**Figure 3:**
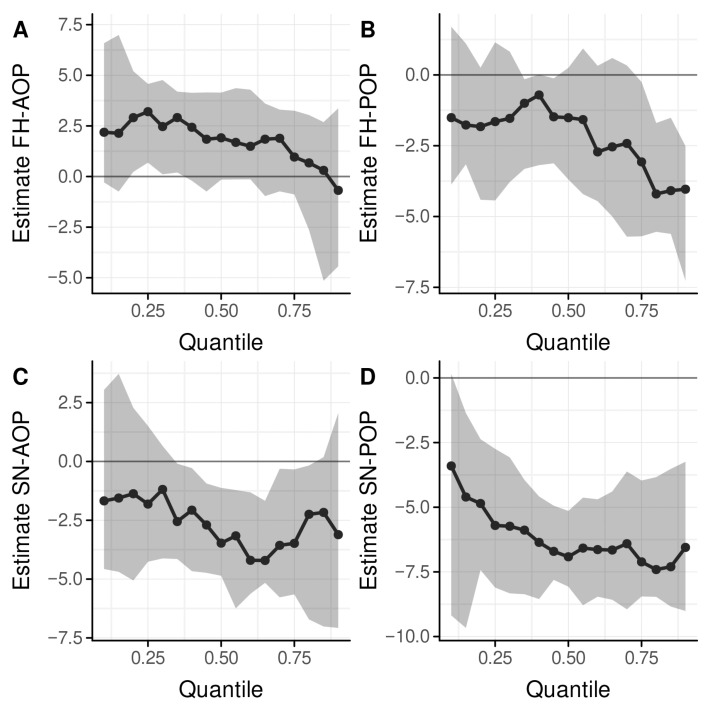
Quantile regression plots showing the sequence of between-group differences (class III–class II) for (**A**) FH–AOP, (**B**) FH–POP, (**C**) SN–AOP, and (**D**) SN–POP from quantiles 10% to 90%, adjusted for age, gender, and vertical pattern (ODI). Adjusted estimated group differences can differ between the quantiles along the x-axis. The dotted line depicts estimates of the difference over the distribution of data; the grey area shows the 95% confidence interval. Note: Statistical significance where 95% confidence interval does not include zero.

The correlations of FH–AOP vs. SN–AOP, FH–POP vs. SN–POP, and APDI vs. Wits appraisal were significant (*p* < 0.01) and yielded r = 0.71, r = 0.68, and r = −0.86, respectively. Table 2 shows estimates for the between-group differences including 95% confidence intervals and the results of ANOVA and quantile regressions at the quartiles. Figure 3 illustrates the quantile regressions of the group differences from the 10th to the 90th percentiles.

### 3.1. Analysis of Variance

FH–AOP did not differ significantly between the class II and class III groups. Contrarily, the means of SN–AOP, FH–POP, SN–POP, and ODI showed significant differences between the class II and class III groups (*p* = 0.004, *p* = 0.006, *p* < 0.001, respectively; Table 2).

### 3.2. Quantile Regression

The quantile regression models showed that age and gender did not differ significantly between the groups. The models estimated an increase in FH–AOP and FH–POP by approximately 1–1.5° for each additional year of age while SN–AOP and SN–POP measurements demonstrated annual changes of ±0.5°.

Significant between-group differences existed for FH–AOP at Q_1_ (*p* = 0.012), SN–AOP at Q_2_ and Q_3_ (*p* = 0.04, *p* = 0.011), FH–POP at Q_3_ (*p* = 0.006), and SN–POP at all 3 quartiles (*p* < 0.001). ODI showed significant between-group differences at Q_1_ (*p* = 0.018), Q_2_ (*p* < 0.001), and Q_3_ (*p* = 0.001).

## 4. Discussion

The allocation of individuals to skeletal groups according to the APDI corresponded to the Wits appraisal measurements. The principle “*steeper plane of occlusion in skeletal class II than in skeletal class III*” was applicable for mixed dentitions. This novel outcome was not only significant at the means but also at the median (SN–POP), the lower quartile (SN–POP), and the upper quartile (FH–POP, SN–POP). Class II POP inclinations exceeded those of the class III group in a range from 1.5 to 3.5° (FH–POP) and 5.1 to 7° (SN–POP). The POP inclinations indicated specific traits of the posterior occlusion and separated skeletal classes II and III in the mixed dentition period. Children aged from 5.6 to 11.7 years showed early stages of adult occlusal plane characteristics over the distribution of data.

The abovementioned principle did not work for FH–AOP because its mean, median, and Q_3_ did not differ significantly between the groups. Conversely, SN–AOP yielded significant between-group differences in means, medians, and upper quartiles, i.e., 2.6–3.5° greater means and quartiles in the class II group. Difficulties in identifying porion and orbitale same as errors from averaging these structures may explain the discrepancy between the FH–AOP and SN–AOP results [20]. Our study’s 95% limits of agreement and ICCs for repeated measurements align with the finding from the literature. Hence, we considered SN-related measurements as more valid than FH-related angles but kept the latter for comparison with Tanaka and Sato’s investigation [6]. The significantly steeper SN–AOP mean, median, and upper quartile indicated a predisposition of the class II group toward increased incisor overbite.

The annual increase in our sample’s FH–AOP and FH–POP implied skeletal alterations such as orbital development or maxillary growth and displacement. These factors clearly affect the FH. On the other hand, the annual changes in SN–AOP and SN–POP were close to zero. This finding indicates a relatively stable cranial base during the investigated period. However, SN rotated clockwise by 0.35° during 11 months in treated 8- to 10-year-old children with skeletal class II [21]. This rotation ceased at age 11 years and resembles our study’s annual changes in SN–AOP and SN–POP. Still, these changes do not explain the salient between-group differences in SN–POP (5–7°; Q_1_, Q_3_) and SN–AOP (2.5–3°; Q_1_, Q_3_).

Garcia Santana et al. [21] did not investigate cranial base changes in children with skeletal class III. Theoretically, a counter-clockwise orientation of the class III cranial base could have added to the difference between both groups but such reasoning requires explicit study. Another aspect of class III malocclusion relates to a typically acute cranial base angle. Yet, the occipital cranial base and not the anterior cranial base showed significant geometric morphometric deformations in class III subjects [22].

Tanaka and Sato had studied the inclinations of FH–AOP and FH–POP in a sample selected from the Burlington growth collection. Having evaluated statistical means only, neither FH–AOP nor FH–POP differed significantly between class II and class III malocclusions at ages 6–7 years and 8–10 years [6]. The different outcome in statistical significance of FH–POP between Tanaka and Sato’s paper and the current study may originate from diverse sample sizes and the APDI values per se. The Burlington class III subsample had remarkably smaller APDI means (11° less class III at age 6–7 years and 8° less at age 8–10 years). On the other hand, the Burlington class II subsamples of the 6–7- and 8–10-year-olds showed less skeletal class II than our sample (1.3° and 2.5° greater APDI means, respectively).

Regarding the vertical growth pattern, the ODI was not a strong effect modifier in our sample. The ODI means of Tanaka and Sato’s study were similar but the present study’s ODIs differed significantly between the normodivergent class II group and the hyperdivergent class III group. Tanaka and Sato’s class III group showed marginally shorter faces. Their ODIs did not differ significantly from those of the class II Burlington subsample at ages 6 and 7 years. However, the between-group difference in ODI became significant in Tanaka and Sato’s 8- to 10-year-olds.

Both investigations probably differ in radiograph quality. The present study exclusively involved digital radiography. Its enhanced imaging could have allowed for more accurate landmark identification than conventional radiography [23]. In addition, we used a slightly different definition of POP for better representation of the sagittal occlusal curve. We traced the distal cusp tip of the averaged maxillary first permanent molar whereas Tanaka and Sato used the midpoint of the same tooth at the level of occlusion.

AOP and POP roughly characterise the curve of Spee. Its initial depth was 0.25 mm in deciduous dentitions and increased significantly after eruption of permanent incisors and permanent first molars to 1.3 mm [24]. Since steep POPs have been observed in permanent dentitions with class II malocclusion [4,6,7,8], accentuated curves of Spee may have impeded the forward growth of the mandible mechanically. Contrarily, flat occlusal curves and flat POPs could facilitate horizontal growth of the mandible [3,6]. In the present study, AOP and POP formed angles by an average of 4.3° (median 5°) in the class II group but 1.3–1.5° at the mean and median in the class III group. The difference in class II and class III mixed dentition curves of Spee also anticipated the specific occlusal characteristics of permanent dentitions.

Various malocclusions emerge in mixed dentitions and may require timely diagnosis [25,26,27]. As proposed by Tanaka and Sato [6], comprehensive orthodontic treatment planning should incorporate the inclination of the maxillary POP. Regarding an aetiologic context between AOP or POP inclinations and class II or class III malocclusions, our study does not shed light on cause and effect. However, the association between sagittal inclinations of AOP or POP and sagittal malocclusions may bear clinical implications. Future investigations should explore the therapeutic effects of changing AOP and POP inclinations in class II and class III mixed dentitions. Decided design and purposeful grinding of functional appliances or focused use of headgears exemplify interceptive interventions, which alter the OP inclination [28]. Such action could counteract development of considerably inclined AOPs and POPs. Hypothetically, alteration of AOP and POP could also enhance orthodontic treatment or its stability. All these aspects require further research.

Methodological restrictions of the present study include the retrospective cross-sectional design, a two-dimensional analysis of three-dimensional structures, errors from averaging bilateral landmarks, which also affect AOP and POP, and a sample of Europeans only. Having used cross-sectional data, the findings exclusively pertain to conditions depicted on the radiograph for a certain point in time. Hence, this study cannot refer to longitudinal aspects, which may affect AOP and POP inclinations because of craniofacial growth, further eruption of teeth, attrition, or remodelling. The quantile regression models included age as covariate without differentiation of certain mixed dentition phases. Lacking distinction of these phases may have influenced the measurements. We assume that the frequencies of these phases were similar in both skeletal groups as age and gender did not show significant between-group differences. Care must be taken before associations from this study are extrapolated to therapeutic alteration of AOP or POP inclinations in mixed dentitions because of sparse longitudinal data and absent interventional evidence.

## 5. Conclusions

In the mixed dentition period, children with skeletal class II showed approximately 3° steeper SN–AOP inclinations and approximately 6° steeper SN–POP inclinations than children with skeletal class III. This divergence represents morphological traits of mixed dentitions, corroborated over the distribution.

The adjusted differences in means and quantiles of this sample did not depend on the vertical pattern.

Longitudinal and 3D data still must confirm the resultant clinical hypothesis of flattening or steepening AOP and POP in mixed dentition class II or class III malocclusions before translating this research perspective to treatment targets.

## Figures and Tables

**Table 1:** Descriptive statistics of the skeletal class II group (n = 61) and the skeletal class III group (n = 60). Age in years, angles in degrees, Wits appraisal in millimetres. Abbreviations: SD standard deviation, IQR interquartile range, Min minimum, Max maximum, Q_1_ 0.25 quartile, Q_3_ 0.75 quartile.

	Group	Mean	SD	Median	IQR	Min	Max	Q_1_	Q_3_
Age	II	8.2	1.5	8	2	5.6	11.7	7	9
	III	8.6	1.5	8.6	2.4	6	11.3	7.5	9.9
APDI	II	72.8	3.8	74	3	59	77	72	75
	III	89.8	3.8	89	5.6	85.3	102	86.5	92
FH–AOP	II	8.6	5.5	8	7.5	−3	21.5	4	11.5
	III	9.9	4.6	10.5	5.5	−1	20.5	7	12.5
FH–POP	II	12.9	5.2	13	7	2	23	10	17
	III	11.1	3.8	11.2	5	2.5	21	8.5	13.5
SN–AOP	II	20.2	5.3	20	7	3.5	31	17	24
	III	17.6	5.3	16.8	6.5	7	30	14.5	21
SN–POP	II	24.5	4.6	25	6	13	32	22	28
	III	19	4.3	19	4.5	5	31	16.9	21
ODI	II	73.1	7.8	74	10	57.5	89	67	77
	III	66.6	8.5	66.5	12.5	27	81	61	73.5
Wits appraisal	II	2.7	2.5	3	3	−3.5	9.5	1	4
	III	−4.3	2.5	−4.5	3.6	−10	4.5	−6	−2.5

**Table 2:** Differences between skeletal classes II and III (estimate = class III—class II) using ANOVA F test (mean) and quantile regressions adjusted for age, gender, and vertical pattern (ODI). Significant *p*-values in bold. Abbreviations: Q_2_ median, Q_1_ 0.25 quartile, Q_3_ 0.75 quartile, CI_0.025_ lower 95% confidence interval bound, CI_0.975_ upper 95% confidence interval bound.

Measurement	Method	Estimate	CI_0.025_	CI_0.975_	*p*-Value
FH–AOP	ANOVA	1.57	−0.34	3.48	0.11
	Q_2_	1.91	0.24	3.85	0.11
	Q_1_	3.2	1.1	4.2	**0.012**
	Q_3_	0.96	−0.5	2.79	0.52
					
SN–AOP	ANOVA	−2.22	−4.35	−0.1	**0.041**
	Q_2_	−3.47	−4.56	−1.17	**0.004**
	Q_1_	−1.81	−3.57	1.22	0.22
	Q_3_	−3.48	−5.35	−0.76	**0.011**
					
FH–POP	ANOVA	−2.4	−4.1	−0.7	**0.006**
	Q_2_	−1.51	−3.31	0.07	0.17
	Q_1_	−1.65	−4.35	0.97	0.21
	Q_3_	−3.07	−5.32	−0.66	**0.006**
					
SN–POP	ANOVA	−6	−7.78	−4.22	**<0.001**
	Q_2_	−6.92	−7.98	−5.16	**<0.001**
	Q_1_	−5.7	−7.51	−3.2	**<0.001**
	Q_3_	−7.11	−8.38	−4.44	**<0.001**
					
ODI	ANOVA	−7.02	−9.97	−4.06	**<0.001**
	Q_2_	−8.55	−10.5	−4.15	**<0.001**
	Q_1_	−6	−10.55	−0.05	**0.018**
	Q_3_	−6.03	−8.5	−3.4	**0.001**

## Data Availability

The datasets used and analysed during the current study are available from the corresponding author upon reasonable request.

## Supplementary Materials

The following supporting information can be downloaded at: https://www.mdpi.com/article/10.3390/jcm14186553/s1, Table S1: Estimates of Ordinary Least Squares Models.

## Abbreviations

AOP	Anterior Occlusal Plane
APDI	Anteroposterior Dysplasia Indicator
FH	Frankfort Horizontal
ICC	Intraclass Correlation Coefficient
POP	Posterior Occlusal Plane
ODI	Overbite Depth Indicator
OP	Occlusal Plane
Q_1_	25% Quartile
Q_2_	50% Quartile, median
Q_3_	75% Quartile
SD	Standard Deviation
SN	Sella-Nasion line
