# Supplementary material for: Anterior and Posterior Occlusal Plane Inclinations Differ Between Class II and Class III Mixed Dentitions—A Retrospective Cross-Sectional Study of a Morphological Characteristic
Source: J Clin Med. 2025 Sep 17;14(18):6553. doi: 10.3390/jcm14186553 (PMC12470786; doi:10.3390/jcm14186553)
Supplement: Supplementary file 1 [file jcm-14-06553-s001.zip › jcm-3823346-supplementary.pdf]

Journal of
Clinical Medicine

Table S1. Estimates of Ordinary Least Squares Models.

		β	SE	p
Adjusted model FHAOP	Intercept	-8.25	4.39	
	Class III - Class II	1.57	0.96	0.106
	Age	10	3.05	0.001
	Gender	0.27	0.88	0.765
	ODI	0.12	0.05	0.034
Unadjusted model FHAOP	Intercept	8.61	0.65	
	Class III - Class II	1.26	0.92	0.174
Adjusted model FHPOP	Intercept	3.54	3.91	
	Class III - Class II	-2.4	0.86	0.006
	Age	12.3	2.71	<0.001
	Gender	0.16	0.79	0.843
	ODI	-0.01	0.05	0.836
Unadjusted model FHPOP	Intercept	12.9	0.58	
	Class III - Class II	-1.79	0.83	0.033
Adjusted model SNAOP	Intercept	17.1	4.9	
	Class III - Class II	-2.22	1.07	0.041
	Age	-1.64	3.4	0.63
	Gender	0.21	0.99	0.835
	ODI	0.06	0.06	0.324
Unadjusted model SNAOP	Intercept	20.2	0.68	
	Class III - Class II	-2.67	0.96	0.006
Adjusted model SNPOP	Intercept	28.1	4.09	
	Class III - Class II	-6	0.9	<0.001
	Age	1.45	2.84	0.61
	Gender	0.08	0.82	0.919
	ODI	-0.07	0.05	0.203
Unadjusted model SNPOP	Intercept	24.5	0.57	
	Class III - Class II	-5.51	0.81	<0.001
